# The New Hall for Operations at the Large Municipal Hospital, Copenhagen

**Published:** 1892-04-09

**Authors:** 


### HOSPITAL CONSTRUCTION.

### THE NEW HALL FOR OPERATIONS AT THE LARGE MUNICIPAL HOSPITAL, COPENHAGEN.

A correspondent writes: At the large Municipal Hospital in Copenhagen, which is generally admitted to be one of the best arranged institutions of its kind, a new operating theatre has just been completed, and as it is in every respect designed in accordance with the most approved principles, a short description of it may interest the readers of this journal. The operation hall is situated on the first floor in the principal wing of the large hospital, and comprises the hall for operations, an ante-room, two preparing rooms, a bath-room with shower-bath for the professors, dressing-rooms and lavatories for the younger doctors, and similar conveniences for the students. There are further efficient arrangements for ventilation, with a fresh-air room in the basement, a heating room and a distribution room for the air. There are also filter chambers for the air before it enters the operation and preparing rooms.

The hall for operations is partly heated by two nickel-plated copper steam stoves in the apartment itself, and partly by a steam stove placed between the double windows. The interior area of the windows will thus act as a heater, and no moisture or steam in the atmosphere of the room will condense on their surface. The preparing or ante-rooms are heated by a nickel-plated copper steam stove in each room, and the different other rooms are also heated by steam.

The fresh air for the ventilation enters the fresh-air compartment in the basement from the large court-yard, being filtered on entering, and, if the outside temperature is below freezing point, the air is warmed to freezing point by means of a steam pipe. A ventilator worked by an electric motor then blows the fresh air through a cast-iron pipe to an air-washer of copper, placed in the heating compartment next to the operation room. In the washer the air passes first through a rain bath and then through a series of "veils" of very finely distributed water. From the air-washer the air enters the heating compartment, where it is heated by means of three copper tubes under the ceiling. In the summer the air can be cooled in the same compartment by letting cold water into the copper tubes.

The air passes from the heating compartment to the distributing room through an upper or a lower aperture in the partition between them. Both apertures can be closed by slides, which are handled from the ante-room to the operating theatre. The warmest air passes through the upper, and the coldest air through the lower aperture. From the distributing room proceed four canals to the filter rooms in the two operation apartments, and to the ante-rooms. The air can be sent to the four rooms mentioned through these four canals. The capacity of the ventilating appliances is calculated at 18,000 cubic feet the hour, but is in reality perhaps fifty per cent. more.

From the operating theatre and from the two ante-rooms the suction of the air takes place, through an upper or a lower aperture.

The operation hall is lighted by four Siemen's regenerative lamps, No. 11.

The operating theatre and the two ante-rooms are fitted with hot and cold water, as are the bath and dressing rooms. In one of the ante-rooms is a copper tank for sterilising the water, holding about 200 litre, with taps both in the ante-room and in the operation hall. The water is made to boil by means of a steam-pipe let into the tank, and can be cooled by cold water passing through the same pipe. In the operating theatre there is a steriliser for the various bandage materials; the disinfection is brought about by steam. At the door of the operating theatre is a steam spray, with a steam valve in a recess in the corridor. The disinfective fluid, which has to be dropped into this spray, is led into it from a tank in the recess. The operating theatre, the ante-rooms, and the air-heating compartment can be cleaned with water from the pipe. All the filters can be taken down and cleaned, and there are reserve filters to be substituted in the meantime. Altogether, every possible precaution is taken to ensure perfect disinfection.

